# P-162. Estimating the Seroprevalence of HTLV-1 in Pregnant Peruvian Women Based on Blood Bank Markers: a Road to Elimination of Mother-to-Child Transmission of HTLV-1

**DOI:** 10.1093/ofid/ofae631.367

**Published:** 2025-01-29

**Authors:** Jorge Robledo, Julianne Meisner, Joseph Zunt, Elsa Gonzalez-Lagos

**Affiliations:** University of Washington, Seattle, Washington; University of Washington, Seattle, Washington; University of Washington, Seattle, Washington; Universidad Peruana Cayetano Heredia, Lima, Lima, Peru

## Abstract

**Background:**

The prevention of mother-to-child transmission (PMTCT) of human T-cell lymphotropic virus type 1 (HTLV-1) is a top priority for HTLV-endemic countries due to its chronicity, lack of treatment and association with the aggressive adult T cell leukemia/lymphoma in comparison to other routes of infection. As of 2024, Japan, Brazil and Chile are few of the countries with national policies regarding antenatal screening. Peru, also an endemic country with around 150,000 to 450,000 HTLV-1 carriers, has limited data in pregnant women and does not have any PMTCT policy in place. By contrast, HTLV-1/2 antibody screening of blood donations has been endorsed since 1997 and national data are available. We aim to leverage these data to estimate the prevalence of HTLV-1 in pregnant women as a first step towards development and implementation of a PMTCT policy in Peru.

Figure 1

**Figure:**
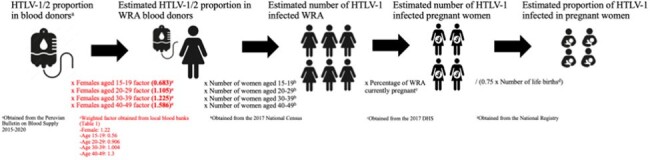

Estimation of number of HTLV-1 infected pregnant women and HTLV-1 seroprevalence in pregnant women in Peru (2017)

**Methods:**

This was an ecological study using public-use data from the Peruvian Bulletin on Blood Supply 2015-2020, the 2017 National Census, the 2017 Demographic and Health Survey (DHS) and the National Registry. The unit of analysis was Peru and its subregions in the year of 2017. To estimate the number of infected women of reproductive age (WRA, 15-49 years), we used census data and a calculated percentage of HTLV-1/2 reactive blood units for this group based on a weighted factor by sex and age (Figure 1, Table 1). Using the DHS percentage of WRA currently pregnant, we estimated the number of infected pregnant women. To obtain the HTLV-1 prevalence for this group, we used the number of life births adjusted for a 9-month period (0.75) as the denominator.

**Figure:**
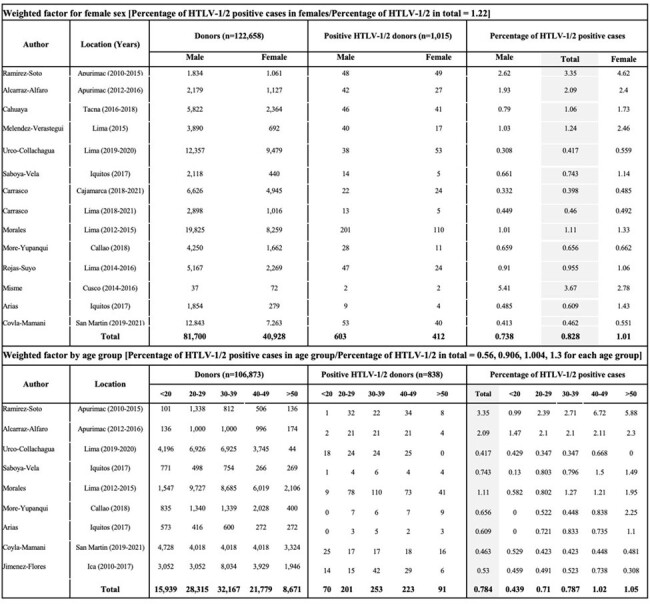

Calculation of weighted factor for HTLV-1/2 seroprevalence by age and sex based on local blood bank data

**Results:**

A total of 359,458 blood units were screened and 2,809 were reactive for HTLV-1/2 (0.78%) (Figure 2). We estimated between 68,456 to 72,978 HTLV-1 carriers who were WRA, of whom 2,463 to 2,773 were pregnant (Figure 3). This is comparable to rates in Japan and Brazil. Our estimated HTLV-1 seroprevalence in pregnant women was 0.61% for the entire country.

**Figure:**
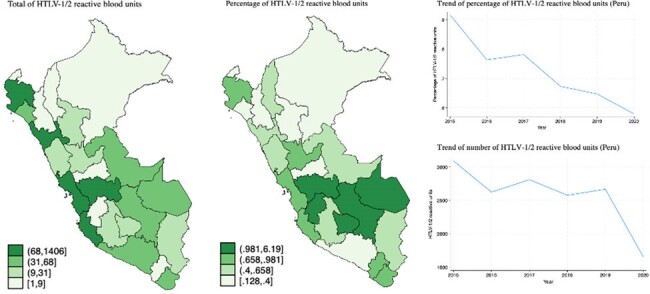

Number and percentage of HTLV-1/2 reactive blood units in 2017 by departments of Peru

**Conclusion:**

Our findings could help guide future strategies for PMTCT, such as universal antenatal screening and assessing the feasibility of formula supplementation for infants born to HTLV-1 positive mothers. Our limitations included the use of one-year data, HTLV-1/2 false-positives in blood donors and not using inverse probability weighting.

**Figure:**
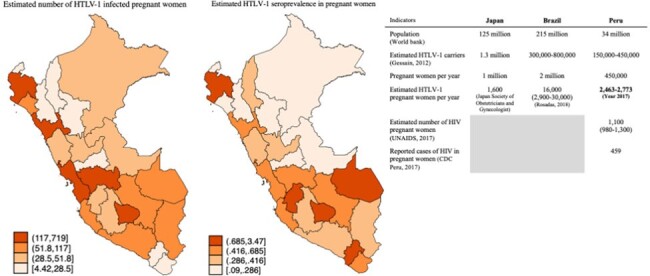

Estimated number and percentage of HTLV-1 infected pregnant women in 2017 by Departments of Peru

**Disclosures:**

**All Authors**: No reported disclosures

